# Controlling Synthetic Cell-Cell Communication

**DOI:** 10.3389/fmolb.2021.809945

**Published:** 2022-01-05

**Authors:** Jefferson M. Smith, Razia Chowdhry, Michael J. Booth

**Affiliations:** Chemistry Research Laboratory, University of Oxford, Oxford, United Kingdom

**Keywords:** control, synthetic biology, communication, stimuli responsive, synthetic cell, artificial cell

## Abstract

Synthetic cells, which mimic cellular function within a minimal compartment, are finding wide application, for instance in studying cellular communication and as delivery devices to living cells. However, to fully realise the potential of synthetic cells, control of their function is vital. An array of tools has already been developed to control the communication of synthetic cells to neighbouring synthetic cells or living cells. These tools use either chemical inputs, such as small molecules, or physical inputs, such as light. Here, we examine these current methods of controlling synthetic cell communication and consider alternative mechanisms for future use.

## Introduction

The construction of an autonomous synthetic cell (SC) from the bottom-up is a formidable task. Hence, rather than creating a single compartment that performs every necessary cellular function, most bottom-up SC research focuses on recapitulating specific cellular hallmarks such as metabolism (Lee et al., 2018; Berhanu et al., 2019), division (Zhu and Szostak, 2009; Kretschmer et al., 2019; Miele et al., 2020; Steinkühler et al., 2020; Dreher et al., 2021), and communication (Lentini et al., 2014; Booth et al., 2016; Lentini et al., 2017; Ding et al., 2018; Niederholtmeyer et al., 2018; Rampioni et al., 2018; Tang et al., 2018; Joesaar et al., 2019; Toparlak et al., 2020; Chakraborty and Wegner, 2021), into simple cell models. Membrane-defined SCs have been developed from liposomes (Stano, 2019), emulsion droplets (Booth et al., 2017a), polymersomes (Rideau et al., 2018), and proteinosomes (Huang et al., 2013) that feature an internal lumen surrounded by a physical boundary comprised of a lipid bilayer or monolayer, amphipathic polymers, or proteins, respectively. The lumen can be filled with enzymes, small molecules, or even a minimal mixture of enzymes and small molecules capable of performing cell-free protein synthesis (CFPS). Membrane-less SCs have also been assembled from coacervates (Mason and van Hest, 2019).

Of the many cellular traits, communication has received significant attention as it offers the promise to interface the various forms of SCs with living cells, or assemble communities of interconnected SCs that perform more complex tasks as a collective. Communication between populations of SCs, or SCs and living cells, has been achieved with liposomes (Gardner et al., 2009; Lentini et al., 2014; Lentini et al., 2017; Tang et al., 2018; Toparlak et al., 2020), droplet networks (Booth et al., 2016; Schwarz-Schilling et al., 2016; Dupin and Simmel, 2019), proteinosomes (Tang et al., 2018; Joesaar et al., 2019), and polymersomes (Niederholtmeyer et al., 2018), and tends to employ two main approaches; Using either 1) membrane-permeable signalling molecules formed *in-situ* from precursors contained within the compartment, through chemical (Gardner et al., 2009) or enzymatic reactions (Lentini et al., 2017; Ding et al., 2018; Rampioni et al., 2018; Tang et al., 2018; Wang et al., 2019; Buddingh’ et al., 2020), or 2) membrane-impermeable signalling molecules encapsulated or formed inside a compartment, and then released into the environment via the insertion of pore-forming proteins (Adamala et al., 2017; Tang et al., 2018; Toparlak et al., 2020) or peptides (Wang et al., 2019) into the membrane. Current cutting-edge applications of SCs, using these mechanisms, include the delivery of therapeutic proteins to tumours *in vivo* (Krinsky et al., 2018), detection and subsequent killing of bacterial cells (Ding et al., 2018), and directed differentiation of neuronal stem cells (Toparlak et al., 2020).

These approaches represent the foundations of how communication between SCs can be achieved, but the processes involved are poorly regulated. Pore-dependent communication using encapsulated signalling molecules often suffers from molecule leakage, either due to the SCs rupturing or leaky expression of the pore proteins (Lentini et al., 2014), while *in-situ* synthesis of signalling molecules often lacks regulation altogether when encapsulated or constitutively expressed enzymes are used (Ding et al., 2018; Rampioni et al., 2018; Wang et al., 2019).

For SCs to become a mature technology with meaningful applications, their activities must be regulated to ensure they function only under the desired context or at the intended time. Hence, we must find better ways to regulate molecular communication in SCs; an area that currently lags behind creating the communication routes themselves. If we can regulate communication with more biologically relevant molecules, SCs could play a role in modulating the microbiome. Alternatively, by controlling communication spatially or temporally with light or temperature, SCs could be applied in various fields, including tissue engineering and biomedicine. Several in-depth reviews covering recent advances in SC communication systems have been published over the last few years (Rampioni et al., 2019; Robinson et al., 2021; Mukwaya et al., 2021). In this review, we describe how SC communication systems have been controlled, and explore alternative parts that might be used to control SC communication in biologically useful ways, as well as spatially and temporally using remotely-controlled stimuli. We focus our attention on liposome-based SCs, but these tools are broadly applicable to other forms of SCs.

## Current Methods of Controlling Synthetic Cells

### Molecular Control of Synthetic Cell Communication

**FIGURE 1:**
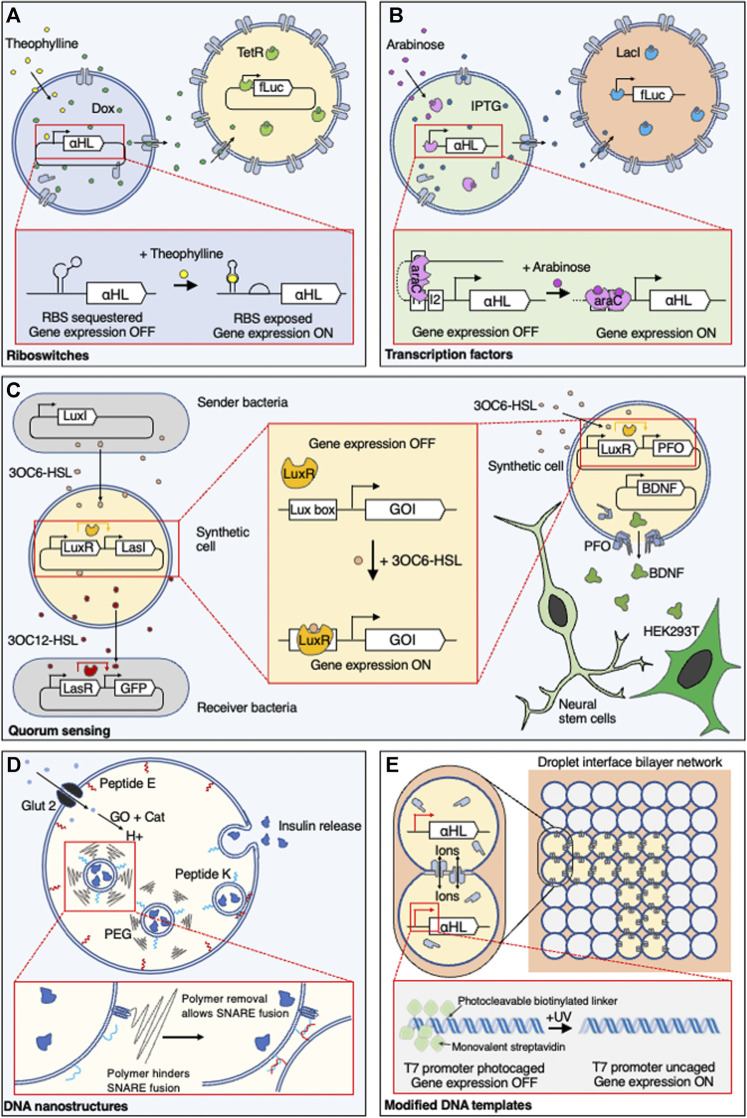
Controlling communication in synthetic cells. Genetically encoded synthetic cell communication systems have been controlled by regulating the expression of αHL using **(A)** small molecule sensitive riboswitches or **(B)** transcription factors. (**C**—left) Transcription factors that recognise acyl-homoserine lactones are typically used to regulate quorum sensing between *E. coli* and synthetic cells or synthetic cells and other synthetic cells, (**C**—right) but they have also regulated expression of a large protein pore, PFO, in synthetic cell communication with mammalian cells. **(D)** Control over communication that does not require genetic control has been demonstrated by using pH-responsive DNA nanostructures and polymers to regulate the fusion of entrapped vesicles with the membrane of a larger vesicle and the release of insulin. **(E)** In contrast to molecular activation, communication between synthetic cells has been initiated using light-activated DNA. 3OC6-HSL, N-3-oxo-hexanoyl homoserine lactone; αHL, alpha-hemolysin; araC, arabinose-sensitive transcription regulator; BDNF, brain-derived neurotrophic factor; Cat, catalase; Dox, doxycycline; fLuc, firefly luciferase; GFP, green fluorescent protein; Glut 2, glucose transporter 2; GO, glucose oxidase; GOI, gene of interest; HEK293T, human embryonic kidney 293T cells; IPTG, isopropyl ß-d-1-thiogalactopyranoside; LacI, lac repressor; LasI, 3OC12-HSL synthase gene; LasR, 3OC12-HSL transcriptional activator; LuxI, 3OC6-HSL synthase gene; LuxR, 3OC6-HSL transcriptional activator; PEG, polyethylene glycol-5000; PFO, perfringolysin O; RBS, ribosome binding site; TetR, Tet Repressor protein; UV, ultraviolet.

Unlike natural cells, SCs do not have complex uptake and signal recognition pathways to import and detect molecular cues. Instead, small molecule-activated communication tends to be achieved by the direct interaction of the molecule with its cognate regulator. Most commonly, this is achieved using membrane-permeable small molecules that diffuse across the SC membrane and activate transcription factors (TFs) or riboswitches to control transcription and translation, respectively (Figures 1A–C).

#### Riboswitch Control

Riboswitches are RNA sequences found in the 5’ untranslated region (UTR) of a gene of interest that couple the binding of a small molecule to the sequestration or release of a downstream ribosome binding site (RBS) (Serganov and Nudler, 2013). In this way, small molecules can control gene expression at the level of translation. Pore-dependent molecular communication in SCs has been controlled using riboswitches, through the theophylline-induced expression of a membrane protein pore, alpha-hemolysin (αHL). αHL is a protein pore from *Staphylococcus aureus* that spontaneously assembles and inserts into lipid bilayers (Thompson et al., 2011). By placing the αHL gene downstream of a theophylline riboswitch, membrane-impermeable signalling molecules, encapsulated inside a SC with a CFPS system, were released following binding of theophylline to the riboswitch, causing the release of the RBS, and expression then membrane insertion of the protein pore. Using this approach, isopropyl ß-d-1-thiogalactopyranoside (IPTG) (Lentini et al., 2014; Adamala et al., 2017) and doxycycline (Adamala et al., 2017) have been released from SCs to activate gene expression in neighbouring *E. coli* (Lentini et al., 2014) or SCs (Adamala et al., 2017) (Figure 1A).

#### Transcription Activators

Bacterial small molecule-based transcriptional control systems have been widely used to control gene expression in SCs. One example is quorum sensing (QS) systems that are involved in natural bacterial communication, which use membrane-permeable small molecules, known as N-acyl-homoserine lactones (AHSLs), that are released from bacteria and enter their neighbours to activate transcription regulators (Whitehead et al., 2001). The well-characterized QS pathway from *Vibrio fischeri* (LuxR) (Fuqua et al., 1994) has been reconstituted into SCs to sense an AHSL and mediate communication between two populations of bacteria that do not usually communicate with one another (Lentini et al., 2017). Cell-free expression of an AHSL synthetase within the SCs was activated by N-3-oxo-hexanoyl homoserine lactone (3OC6-HSL), produced by a population of bacteria, which initiated the synthesis and release of N-3-oxo-dodecanoyl homoserine lactone (3OC12-HSL), and activated reporter gene expression in a secondary population of bacteria (Figure 1C). 3OC6-HSL has also been used to initiate the expression of the large protein pore perfringolysin O inside SCs to release *in-situ* synthesized brain-derived neurotrophic factor (BDNF), which subsequently initiated differentiation of co-incubated neural stem cells (Toparlak et al., 2020) (Figure 1C). An alternative pathway, based on the EsaR repressor protein, was used to activate the release of glucose from liposome-based SCs to communicate with co-incubated proteinosome-based SCs (Tang et al., 2018).

Other bacterial transcription factor (TF) systems used within SCs include the lac repressor (LacI) and arabinose-sensitive transcription regulator (araC), which control expression of the lac and arabinose operons and are commonly used for controlled expression in *E. coli*. Placement of LacI or araC operators upstream of a gene of interest has allowed IPTG and arabinose to initiate communication between emulsion droplet-based SCs (Schwarz-Schilling et al., 2016) and between different populations of liposome-based SCs (Adamala et al., 2017) (Figure 1B).

#### Other Molecular Activators

The addition of alternative activator molecules such as glucose (Chen et al., 2018; Tian et al., 2018; Gobbo et al., 2018; Wang et al., 2019; Li et al., 2020), ATP (Buddingh’ et al., 2020), or ions (Gardner et al., 2009) have also been shown to initiate communication between SCs and living cells. SCs have been produced that could release insulin in response to hyperglycemic levels of glucose and lower the blood glucose levels in a mouse model (Chen et al., 2018). Glucose was taken up via a glucose-specific transporter and converted to hydrogen peroxide by glucose oxidase (GO). The subsequent decrease in the internal pH caused the fusion of internally held vesicles containing insulin to the membrane of the SCs (Chen et al., 2018) (Figure 1D). Glucose has also been used to activate SCs patterned into tissue-like colonies using magnetic (Li et al., 2020) and acoustic (Wang et al., 2019) manipulation. In both examples, glucose was used to initiate the formation of hydrogen peroxide in one population of SCs, which subsequently initiated a fluorescent output in neighbouring cells or cell death in neighbouring mammalian cells. Communication between SCs and bacteria has also been controlled by a change of external pH, which triggered a formose reaction that produced a QS analogue inside liposomes and subsequently activated bioluminescent signalling pathways in *Vibrio harveyi* (Gardner et al., 2009).

A synthetic “predator-prey” interaction was developed between two populations of SCs by adding the membrane-permeable small molecule coelenterazine to initiate intracellular bioluminescence. Light responsive proteins iLID and Nano, in the respective “predator” and “prey” SCs, subsequently adhered together to initiate Ca^2+^ transfer through αHL, causing lysis of the prey SC (Chakraborty and Wegner, 2021).

Control of communication between non-liposomal compartments has been achieved by the addition of glucose to coacervates (Tian et al., 2018) and proteinosomes (Gobbo et al., 2018). The addition of ssDNA, across semi-permeable membranes, has also been used to initiate DNA strand-displacement (DSD) reactions between proteinosomes (Joesaar et al., 2019).

### Physical Control

While small molecule activation is limited to those that can penetrate the SC membrane, physical stimuli such as light, temperature, acoustics, and magnetism can all easily pass through any SC membrane. An additional advantage of using physical stimuli to activate SCs is they can be applied spatiotemporally. Work to date has entirely focused on controlling SC communication with light, which has the advantage of being a biorthogonal, tuneable, and remotely controlled signal.

Light-activated gene expression has been used to spatially control neuron-like communication between SCs formed of aqueous droplets, 3D-printed and bound through lipid bilayers (Booth et al., 2016). This was achieved by sterically blocking the T7 RNA polymerase promoter with seven biotins, each bound to a monovalent streptavidin, conjugated to the DNA through 2-nitrobenzyl photocleavable groups. Ultraviolet (UV) light was used to express αHL in individual SCs (Booth et al., 2017b) or patterned pathways through the droplet networks (Booth et al., 2016), which allowed movement of ions between only the activated SCs (Figure 1E). Optical tweezers have been used to controllably bring together SCs in a targeted manner, followed by the use of a laser to fuse specific SCs (Bolognesi et al., 2018). This spatiotemporal fusion initiated expression of a fluorescent protein. Light-activated communication was also demonstrated between sender-receiver proteinosomes, using photocleavable DNA templates that could release ssDNA through semi-permeable membranes to initiate DSD reactions in neighbouring SCs (Yang et al., 2020).

## Alternative Parts for Controlling Communication

Although the work highlighted above provides a foundation for controlling SC communication with a variety of mechanisms, the approaches are not without their flaws. The theophylline riboswitch used to control αHL expression is leaky; expression occurs even in the absence of theophylline. Expression of non-selective pores limits the longevity of SC activity, as small molecules required for transcription and translation are released along with the membrane-impermeable signalling molecule, and the QS receiver proteins that have been shown to work in SCs both recognise the same molecule. Several technologies, including stimuli-responsive pores and opto-/thermo-genetics, have been underexplored in SC research to date but could offer tighter and more application-specific control over SC communication. Here, we highlight some underutilised technologies that might be useful tools in future SC communication efforts.

### Protein Pores

Most current work on pore-dependent communication has employed wild-type αHL, however, other natural proteins, such as Outer membrane protein F (OmpF) (Nikaido, 1994) and the Mechanosensitive channel of large conductance (MscL) (Sukharev et al., 1997), or *de novo* designed pores, assembled from protein (Scott et al., 2021; Vorobieva et al., 2021) or DNA (Burns et al., 2013a; Burns et al., 2013b; Diederichs et al., 2019; Thomsen et al., 2019; Fragasso et al., 2021; Lanphere et al., 2021) building blocks, have also been shown to permeabilise membranes. These pores each have different sized channels, membrane preferences, and hence allow the passage of different molecules, although most are still non-selective. By introducing stimuli-responsive peptide motifs or small molecules at various positions inside these pores and channels, via genetic or chemical means, their activity can be regulated with externally controlled stimuli. Gateable pores could therefore be used for targeted or context-dependent release of small molecules.

#### pH and Metal Ions

pH-controlled gating has mostly been demonstrated using OmpF, specifically to gate polymersome membrane permeability. pH-responsive plugs comprised of an amphipathic peptide (Edlinger et al., 2017) or hydrazine molecular cap (Einfalt et al., 2015) have been appended within the entrance of OmpF to regulate flux through the pore. A similar feat has also been achieved via the introduction of six histidines into loops within OmpF, which restricted flux through the pore depending on their protonation state (Ihle et al., 2011). Tetra- or penta-histidine motifs have also been introduced into the pore of αHL, but in this case, the pores were tightly blocked through the binding of divalent metal ions, and subsequently unblocked by chelating the metal ions (Russo et al., 1997; Booth et al., 2019; Alcinesio et al., 2021). Small molecules with pKa’s between 5–8 have also been inserted into the hydrophobic gate of MscL to render it pH-responsive rather than pressure-responsive (Koçer et al., 2006). pH-responsive pores might find use in acidic environments, such as the tumour microenvironment, to release immunomodulators and recruit immune cells.

#### Temperature

Repeats of a temperature-sensitive peptide have been genetically inserted into αHL (Jung et al., 2006). These peptides were soluble below 40°C and restricted current flow through the pore. However, when heated above this temperature they aggregated and enabled higher flux through the pore.

#### Light

**FIGURE 2:**
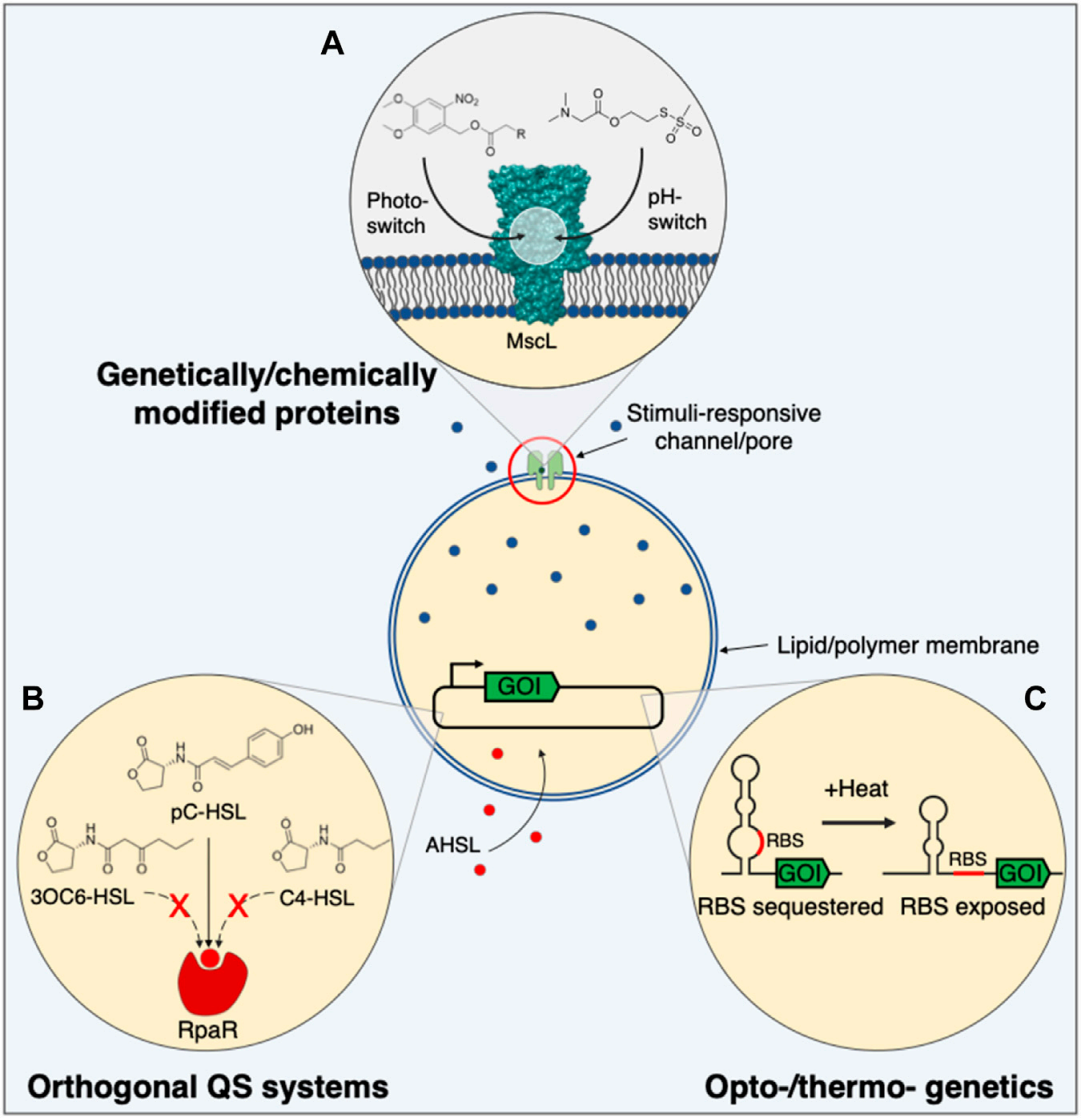
Alternative tools for controlling synthetic cell communication. **(A)** Gateable pore or channel proteins engineered with stimuli-responsive moieties might be used to control the delivery or release of membrane-impermeable signalling molecules in a spatiotemporal or context-dependent manner. **(B)** Orthogonal quorum sensing systems that recognise different acyl-homoserine lactones, and with greater stringency, could diversify the molecules that synthetic cells utilise to regulate gene expression. **(C)** Regulating gene expression inside synthetic cells with temperature or light might offer greater user-defined control over synthetic cell communication for more widespread applications. 3OC6-HSL, N-3-oxo-hexanoyl homoserine lactone; AHSL, acyl-homoserine lactone; C4-HSL, N-butanoyl-l-homoserine lactone; GOI, gene of interest; MscL, mechanosensitive channel of large conductance; pC-HSL, para-coumaroyl-homoserine lactone; QS, quorum sensing; RBS, ribosome binding site; RpaR, pC-HSL transcription regulator.

Light-switchable nanovalves have been developed by installing photosensitive small molecules inside pores and channels (Figure 2A). Conjugation of azobenzene or spiropyran adducts within an αHL pore (Loudwig and Bayley, 2006; Chandramouli et al., 2016) or MscL channel constriction (Koçer et al., 2005; Iscla et al., 2013) reversibly enabled or restricted molecule flux across a membrane through UV and blue light-triggered isomerisation. MscL channels can also be irreversibly opened using light, by installing 6-nitroveratryl caging groups (Koçer et al., 2005). In addition to light-responsive pore blockades, light-regulated insertion of αHL has also been realised by N-terminal fusion of a photoactive protein (Ui et al., 2012) or photocaging residues involved in prepore formation with a 2-bromo-2-(2-nitrophenyl)-acetic acid group (Chang et al., 1995), to switch-on penetration of the pore into lipid bilayers. Light-responsive pores could be used to control the release of small molecules and ions, and might be interfaced with brain tissue to spatially activate neuron signalling without the need for genetic manipulation, unlike optogenetics.

These responsive variants of well-defined protein pores/channels offer a strong foundation towards fully controllable molecular delivery, but computationally or *de novo* designed transmembrane pores may offer more options in the future. Their pore size can be tuned by design (Xu et al., 2020; Lanphere et al., 2021), gated by simple base pairing rules (Burns et al., 2016; Arnott and Howorka, 2019) and there are already a variety of modifications that can be incorporated during solid-phase peptide and nucleic acid synthesis to introduce stimuli-responsive properties.

### Quorum Sensing

Of the many QS systems known, only the well characterised receiver proteins LuxR (Lentini et al., 2017; Toparlak et al., 2020) and EsaR (Ding et al., 2018; Tang et al., 2018) have been successfully used in SCs to date. Despite their different modes of action, transcription activation and repression respectively, both systems recognise 3OC6-HSL. Consequently, it would be difficult to control multiple populations of SCs independently due to signal crosstalk or to distinguish between different species of bacteria. To overcome this, additional QS receiver proteins are required, particularly those that recognise more diverse, physiologically relevant AHSLs. 3OC12-HSL, used in *Pseudomonas aeruginosa* QS, has been detected using a cell-free lasR circuit (Wen et al., 2017), however, the cognate ligand varies from 3OC6-HSL only in the length of the acyl chain. BjaR and RpaR proteins on the other hand activate gene expression in response to branched AHSLs (Lindemann et al., 2011) and aryl homoserine lactones (ArHSLs) (Schaefer et al., 2008) respectively, and could be used to assemble communicating populations of SCs that work orthogonally to SCs that recognise linear chain AHSLs (Scott and Hasty, 2016; Kylilis et al., 2018; Du et al., 2020) (Figure 2B).

### Modulating Gene Expression

Gene expression inside SCs tends to be regulated with small molecule-responsive TFs (Adamala et al., 2017; Lentini et al., 2017; Tang et al., 2018; Dupin and Simmel, 2019; Garamella et al., 2019; Toparlak et al., 2020; Garenne et al., 2021). While these TFs enable SCs to sense environmental cues, some of which are biologically significant, a lack in the diversity of readily exploitable TFs and the requirement that molecules recognised by these proteins be membrane-permeable or able to cross the membrane through a protein pore reduces the versatility of this platform. An underexplored area in SC regulation is the control of gene expression with physical stimuli, for instance, light or temperature. The use of physical stimuli resolves some of these issues surrounding the regulation of gene expression with small molecules and would enable synthetic cells to be used as tools that could be remotely activated using an external, user-controlled stimulus with high spatial precision.

#### Light

Optogenetic activation of αHL expression from light-activated DNA templates has already been used to establish neuron-like communication between networks of droplet-based SCs (Booth et al., 2016), but other light-activated DNAs have also been described. Nitrobenzyl groups installed into the DNA backbone of a plasmid was shown to repress transcription inside SCs until their removal with UV light (Schroeder et al., 2012), while azobenzenes placed in the T7 promoter can offer reversible control over CFPS with UV and visible wavelengths of light (Venancio-Marques et al., 2012; Kamiya et al., 2015). One caveat to the current light-activated DNA templates, and most other synthetic light-activated systems on offer, stems from the use of UV light, which can be cytotoxic at high doses and has little tissue penetration (Olejniczak et al., 2015); therefore, there is a need for light-activated systems that respond to longer wavelengths of light. Alternative optogenetic systems such as light-activated TFs (Jayaraman et al., 2018) or two-component systems (Zhang et al., 2020) can also be used to control cell-free gene expression with more red-shifted wavelengths of light, and other light-activated systems that work well in bacteria may transition into CFPS (Hartmann et al., 2020), although this has yet to be realised. Light-responsive gene expression could be used to controllably produce and release small molecules or proteins and might be applied in tissue engineering efforts to spatially control morphogen gradients and direct tissue differentiation.

#### Temperature

Due to the improved tissue penetration of heat compared to light, temperature-responsive gene expression in SCs may prove useful for *in vivo* communication applications. To this end, temperature-sensitive RNA thermometers that sequester a RBS until a threshold temperature is reached and the secondary structure denatures (Neupert et al., 2008; Kortmann et al., 2011; Sen et al., 2017) (Figure 2C), or thermally-regulated TFs that couple temperature-induced changes in protein folding to their ability to regulate gene expression (Piraner et al., 2017) might be activated remotely using mild hyperthermia and focused ultrasound.

## Discussion

Early SC communication studies used non-physiologically relevant small molecules such as theophylline to activate gene expression. A high concentration of the molecule was required to activate gene expression, and the molecule needed to overcome a membrane barrier to regulate a leaky riboswitch. The introduction of QS systems into SCs improved upon this, creating a more biologically significant and tightly controlled SC communication platform that also allowed SCs to be interfaced with bacterial cells via the detection and production of membrane-permeable AHSLs. However, both QS systems used to date recognise 3OC6-HSL, limiting the versatility of this control mechanism. More recently, SCs have been engineered to respond to other physiologically relevant stimuli, such as glucose concentration, or technologically useful stimuli, such as light, representing further progress towards controlling communication in more biologically and medically useful ways. However, SC communication is yet to be a truly disruptive technology.

To fully realise the potential of SCs in biology and medicine, the existing toolbox and design of SC communication need to be reconsidered. Riboswitches need to be designed for more relevant molecules, something that was recently shown to be possible by the creation of histamine responsive SCs (Dwidar et al., 2019). QS systems must be diversified to establish SCs capable of sensing and responding to more complex populations of bacterial cells, although this may be somewhat challenging as a QS system’s activity isn’t always conserved when transferred from bacteria to cell-free systems (Lentini et al., 2017). Also, stimuli-responsive technologies that respond to pH, temperature or light need to be more readily adopted to apply SCs in a biomedical context and regulate the communication in a spatially controlled manner. Using these improved tools, SCs might be engineered to release signalling molecules inside environments with an acidic pH, such as inside tumours or endosomes, or controllably release neurotransmitters to stimulate neurons without the need for intrusive methods.
